# Hosting an Educational Careers Day Within the Virtual Paradigm: The Neurology and Neurosurgery Interest Group Experience

**DOI:** 10.7759/cureus.21162

**Published:** 2022-01-12

**Authors:** George E Richardson, Conor S Gillespie, Soham Bandyopadhyay, Emma J Norton, Jigi M Joshi, Orla Mantle, Catinca Ciuculete, Armin Nazari, John Ong, Ajitesh Anand, Jay Park, Rosaline De Koning, Setthasorn Zhi Yang Ooi, Joshua Erhabor, Harmani K Daler, Bailint Borbas, Zeluleko Sibanda, Illectra Lerou, Alvaro Y Touzet, Phil Mcelnay, Suzanne Murray, Peter J Hutchinson, Alistair Jenkins

**Affiliations:** 1 School of Medicine, University of Liverpool, Liverpool, GBR; 2 Oxford University Global Surgery Group, University of Oxford, Oxford, GBR; 3 Division of Anaesthesia, University of Cambridge, Cambridge, GBR; 4 Gastroenterology, West Suffolk NHS Foundation Trust, Bury St Edmunds, GBR; 5 School of Medicine, University of Edinburgh, Edinburgh, GBR; 6 School of Medicine, Kings College London, London, GBR; 7 School of Medicine, Queen’s University Belfast, Belfast, GBR; 8 School of Medicine, University of Dundee, Dundee, GBR; 9 School of Medicine, University of Brimingham, Birmingham, GBR; 10 School of Medicine, University of St. Andrews, St. Andrews, GBR; 11 Medical Sciences Division, University of Oxford, Oxford, GBR; 12 School of Medicine, Cardiff University, Cardiff, GBR; 13 School of Medicine, University of Exeter, Exeter, GBR; 14 School of Medicine, University of Lancaster, Lancaster, GBR; 15 School of Medicine, University of Plymouth, Plymouth, GBR; 16 School of Medicine, University of Keele, Keele, GBR; 17 School of Medicine, University of Leeds, Leeds, GBR; 18 School of Medicine, University of Manchester, Manchester, GBR; 19 Medall, Belfast, GBR; 20 Neurosurgery, Society of British Neurological Surgeons, London, GBR; 21 Clinical Neurosciences, Addenbrooke's Hospital & University of Cambridge, Cambridge, GBR; 22 Neurosurgery, Royal Victoria Infirmary, Newcastle upon Tyne, GBR

**Keywords:** covid-19, virtual, conferences, neurosurgery, medical education

## Abstract

Introduction: To explore our experience of hosting the 10^th^ Annual Neurology and Neurosurgery Interest Group-Society of British Neurological Surgeons (NANSIG-SBNS) Neurosurgery Careers Day, held virtually for the first time.

Methods: Reflective feedback and review of an international, virtual neurosurgery careers day. The authors reflect on the logistics of organizing the event, and the pre- and post-event feedback provided by delegates. Recommendations have been made on how to successfully host a virtual event. The key themes that permeated the event have been outlined and discussed in the context of the feedback received.

Results: The event was attended by 231 delegates from 20 countries worldwide. Knowledge of neurosurgery as a career and the application process increased after attending the careers day (4.27/5 to 4.51/5, p=0.003 and 3.12/5 to 4.31/5, p<0.001 respectively). The key themes identified from the event include attendance, networking, and education. Qualitative feedback was positive and indicated a positive perception of the careers day.

Conclusions: The future of educational events is unclear, and a hybrid approach is recommended to retain the benefits of the online space when in-person events eventually return.

## Introduction

The Society of British Neurological Surgeons (SBNS) is the UK’s nationally recognized medical association for neurosurgeons [1]. One of the key focuses of the organization is education and training. To further this aim, the SBNS is partnered with the Neurology and Neurosurgery Interest Group (NANSIG), a medical student and a junior doctor-led national neuroscience organization. Together, the NANSIG and SBNS have delivered an annual neurosurgery careers day for the past nine years. The key objective of this annual event has been to educate medical students and junior doctors about how to pursue a future career as a neurosurgeon in the UK. January 2021 marked the 10th annual NANSIG-SBNS Neurosurgery Careers Day and was the first time the event had been hosted virtually.

The coronavirus disease 2019 (COVID-19) pandemic has had a significant impact on many aspects of life [2]. At the time of writing, there have been 12 million cases and 170,000 deaths from COVID-19 in the UK [3]. During the initial waves of COVID-19, the government implemented social distancing and restrictions to in-person events [4-5]. In addition to the closure of higher education institutions, all in-person conferences were canceled, resulting in a significant disruption to medical education within the UK [6]. In response, event organizers adapted and many events were shifted to the virtual space [7-8]. The proliferation of the virtual paradigm in education has resulted in many organizations traversing uncharted territory.

The aim of this article was to delineate our experience of the NANSIG-SBNS 10th Annual Neurosurgery Careers Day. Furthermore, by reflecting on delegate feedback, we have sought to identify the strengths and negatives of a virtual careers day.

## Materials and methods

Event format

The 10th Annual NANSIG-SBNS virtual Neurosurgery Careers Day was hosted on the 30th of January 2021. The event was free to attend, however, we suggested that an accompanying donation be made to the charity SHOUT, a mental health crisis charity and texting service (https://giveusashout.org/). In order to sign up, delegates were required to provide a valid email address. The only pre-requisite for attendance was access to the online Zoom platform [9]. Zoom was chosen as it was felt most attendees would be familiar with the software given its widespread use in educational institutions, and the availability of features such as breakout rooms. By partnering with E-BrainTM and using their Zoom account we were able to accommodate up to 1000 delegates at the event. Seven days prior to the careers day, a practice event was carried out with the team to troubleshoot any technical issues.

Abstracts for neuroscience-based research were open to international submissions. The online platform MedAll was used to host the poster presentations (https://medall.org/). This was because MedAll is an established organization that has a track record of hosting successful events featuring presentation galleries. From the submissions, five oral presentations and 10 flash poster presentations were selected by dual independent screeners.

In addition to the main speaker program, we hosted curriculum vitae (CV) clinics throughout the day using Zoom breakout rooms, where privacy could be maintained. To attend, delegates were required to register their interest one week in advance. Those who signed up were asked to complete and return a CV document based on the Neurosurgery Speciality Trainee (ST1) Person Specification requirements for national selection in the United Kingdom. Nine neurosurgeons, ranging from trainees to consultants hosted the clinics, and participants were given a 10-minute time slot where they would have a one-to-one discussion with the surgeon to discuss their CV, with priority afforded to foundation doctors and final year medical students. To facilitate the smooth running of the event, a team of 21 NANSIG members was involved in hosting the proceedings.

Information dissemination 

Utilizing the NANSIG social media and email channels, we disseminated a sign-up link using Google Forms prior to the event (Google LLC). The decision to use Google Forms was based on ubiquitous access, ease of dissemination, familiarity with the platform, and that it was free to use. The NANSIG social media channels included Facebook, Twitter, and Instagram accounts. Additionally, the event sign-up was shared using the monthly NANSIG email newsletter. Finally, the SBNS also shared the careers day details using their newsletter.

Data collection

The pre- and post-event questionnaires were constructed by an iterative process of feedback and revision by the event lead (C.S.G) and the NANSIG core committee. The pre-event delegate questionnaire was hosted using Google Forms. Again, this was chosen due to ease of access to the platform. The post-event delegate questionnaire was hosted using the MedAll platform. This was chosen due to the feature of allowing delegate questionnaires to be issued once the feedback form had been completed, thus incentivizing participation. The data were entirely anonymous and by completing the questionnaires, delegates consented to use for research purposes. This was clearly highlighted to delegates prior to completing the forms. Data were stored on a secure computer and was accessible by two authors only (C.S.G and G.E.R). The questions included on pre- and post-event feedback forms are provided in Appendices 1 and 2.

Statistical analysis 

Statistical analysis of pre- and post-event feedback was conducted using R (version 1.4.1). Questions on the event feedback forms were scored using a five-point Likert scale. Statistical analysis was carried out using an independent samples t-test.

## Results

Event attendance and representation

**Figure 1:**
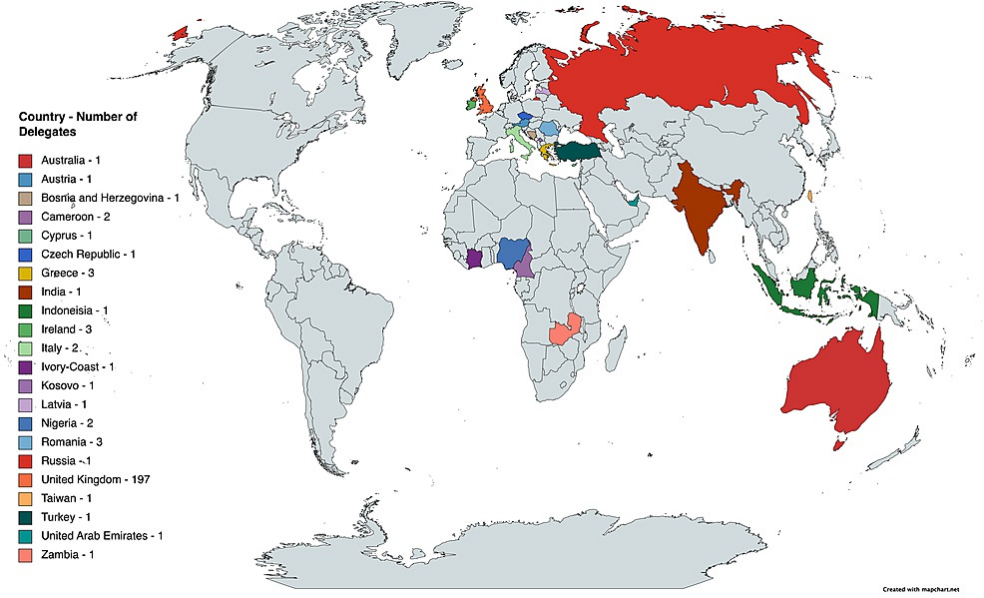
World map showing nation of origin for delegates of the neurosurgery careers day. A key is provided demonstrating which country delegates attended from and the number of attendees from that nation. This map was produced using online software (www.mapchart.net).

**Figure 2:**
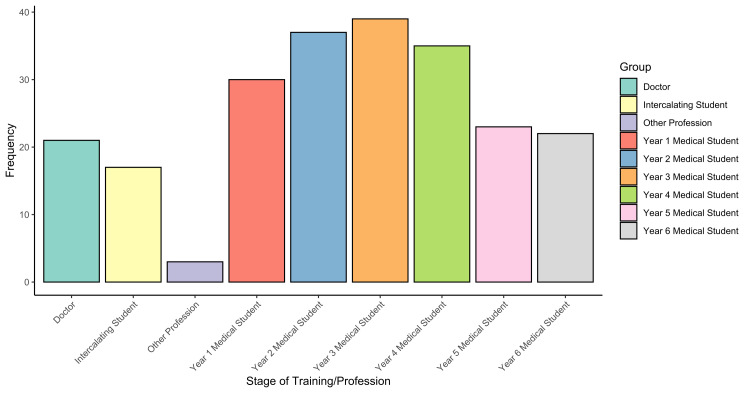
Bar chart demonstrating the stage of training/occupation of delegates of the careers day.

The careers day attracted over 230 attendees, from over 20 countries and four continents across the globe. The worldwide distribution of delegates is highlighted in Figure 1. Attendees were varied in their stage of training, which is summarized in Figure 2.

Based on pre- and post-event feedback, there was a significant increase in agreement with the following statements: ‘I think women are well represented in neurosurgery events’ and ‘I think ethnic minority groups are well represented in neurosurgery events’ (2.54/5.00-4.35/5.00, p<0.001 and 2.86/5.00-4.02/5.00, p<0.001 respectively). Despite the already high levels of interest in neurosurgical careers prior to the event, there was a significant increase highlighted in delegate feedback (4.27/5.00-4.51/5.00, p=0.003). Almost all participants stated an interest in attending further virtual events (4.66/5.00).

Networking and education

Attendees of the CV clinics provided overwhelmingly positive accounts of the experience, as demonstrated by the qualitative feedback in Table 1. Despite efforts to encourage networking in CV clinics, post-event feedback identified that delegates believed virtual events did not provide much chance to network (3.14-3.68, p<0.001).

**Table 1:** Qualitative feedback from CV clinics. […] = name removed.

CV clinic qualitative feedback
“It was awesome and didactic.”
“Was very helpful, a privilege to chat one-on-one with a neurosurgeon.”
“Really helpful answers and really enjoyed the conversation. Very grateful for an honest discussion of the training pathway and applications in general.”
“It was very useful to speak to […], especially given she has only just gone through the application cycle and so has a fresh perspective on everything.”
“Excellent choice of speakers, program was excellent; the idea of breakout rooms to have parallel sessions running was amazing!”

Comparing pre-conference and post-conference feedback, a significant increase in knowledge of neurosurgery as a career was noted (3.12/5.00-4.31/5.00, p<0.001). This was also true of attendees’ awareness of the intricacies of the UK neurosurgical application process (2.91/5.00-4.30/5.00, p<0.001). A full summary of our results can be found in Table 2.

**Table 2:** Pre- and post- event feedback, where available. *1= Not at all interested, 5= Very interested; **1= Not very knowledgeable, 5= Excellent; †1= Non-existent, 5= Excellent; ‡1= Completely disagree, 5= Completely agree

Question	Pre-conference mean score (SD) (n=231)	Post-conference mean score (SD) (n=227)	p value
How interested are you in a career in neurosurgery*	4.27 (0.85)	4.51 (0.71)	0.003
My knowledge of neurosurgery as a career is**:	3.12 (1.03)	4.31 (0.65)	<0.001
My knowledge of the neurosurgical application process in the UK is†:	2.91 (1.20)	4.30 (0.66)	<0.001
‘I think women are well represented in neurosurgery events’‡	2.54 (1.01)	4.35 (0.84)	<0.001
‘I think ethnic minority groups are well represented in neurosurgery events’‡	2.86 (1.00)	4.02 (1.00)	<0.001
‘I think virtual neurosurgery careers days are not as good as in-person ones’‡	2.67 (1.21)	3.00 (1.20)	0.013
‘There is not much chance to network at virtual neurosurgery events’‡	3.14 (1.10)	3.68 (1.12)	<0.001
‘The speakers and program were well organized’‡	N/A	4.63 (0.53)	N/A
‘The conference program was diverse and catered to a variety of interests’‡	N/A	4.45 (0.78)	N/A
‘I would attend another virtual neurosurgery careers day from NANSIG’‡	N/A	4.66 (0.62)	N/A

## Discussion

Increasing attendance

A notable benefit of the shift to virtual events has been the increase in ease of access to educational content [7]. This is true especially for individuals from low-and-middle-income countries (LMICs) [10-11]. Previously, geographical barriers were pervasive in the exclusion of these delegates [8]. With 36% of attending countries being LMICs compared to 0% in previous years, our event demonstrates how virtual events -- especially those that are free -- can help obviate such issues. However, higher costs of data, language barriers, slower connection speeds, and gender gaps in access to technology may now represent the impasse in efforts to increase attendance opportunities for prospective LMIC delegates [12].

Online events make attendance much easier for speakers. Internationally renowned professors and clinicians can deliver talks with ease due to the removal of barriers such as travel costs. Our event featured keynote presentations from the editor-in-chief of a high-impact factor journal, an eminent professor of neurosurgery, and a world-leading innovator in surgical simulation. Inviting these individuals would have been much more difficult to facilitate had it not been for the ease of attending online events [13]. Additionally, talks may be pre-recorded, with the added benefit of using these as a contingency plan if technical issues arise on the day. Moreover, the event itself may be recorded. The 10th Annual Careers Day was captured in its entirety and is available to view, free of charge, for anyone who was unable to attend on the day on the NANSIG YouTube channel (https://youtu.be/gtIgaj4pqkI).

The benefits of increased attendance at events are juxtaposed against the increased demand placed on larger organizations that already attract large numbers of delegates. Despite the relative reduction in costs when organizing an online event, there can still be a sizable overhead when accounting for hosting platforms [13]. For the 10th Annual Careers Day, a budget of approximately £500 was used. However, many of the requirements were accessed at a lower or no cost, by sponsorship and discussion with providers. Proprietary platforms can range widely in both affordability and effectiveness, and this cost is worsened by an expectation for online events to be free and well organized [13]. Awareness is needed from attendees that virtual events cannot always be run free of charge, whilst organizers should be encouraged to disclose their pricing rationales so that delegates can be confident in the fairness of ticket pricing. Larger organizations may be more adversely affected, as they will require more resources to accommodate a higher number of attendees, especially if they have also lost considerable revenue from the cancellation of in-person events across 2020 and 2021.

Feedback from the careers day demonstrated that delegates felt that both women and ethnic minorities were well represented. This finding is important, as it highlights the potential of virtual events to advocate for minority groups. Nonetheless, further work is essential to ensure that groups who have been historically dissuaded from entering neurosurgery are provided adequate opportunity for involvement [14-15].

Event networking

Creating future relationships is a key benefit of attending educational events. The CV clinics were envisioned as a way for delegates to get individualized, useful feedback for their potential applications. Moreover, the one-to-one aspect gave a more personal feeling to the day and facilitated networking between students and clinicians. Creating future relationships is a vital aspect of improving access to neurosurgery -- and indeed other specialties -- for prospective students [16-17].

Although the CV clinics were successful in facilitating networking between individuals and clinicians, it is likely that this attitude was directed more towards inter-student networking opportunities. During most online events, delegates are often instructed to ensure their cameras and microphones are turned off to limit disruptions. Whilst this is done for good reason, it undoubtedly contributes to the feeling of isolation one might experience whilst in attendance [18]. A possible way to negate this would be to host dedicated networking sessions during an online event. Such workshops may be heterogeneous in nature and may take the form of small group discussions, live Q&A sessions, or chatrooms [19].

Educational impacts

Despite the sizable shift towards a virtual educational setting, there remain barriers to e-learning. One barrier to the adoption of e-learning that has previously been identified is the required technical skill [20]. Such problems were encountered first-hand during the careers day, and on reflection, it is clear how this can cause a reluctance to adopt virtual learning approaches. Our problems arose when two of our key speakers were unable to log onto the Zoom meeting. Troubleshooting these issues was compounded further by having to do so remotely [21]. Eventually, our technical issues were resolved, and the day carried on without any further major disruption. The frustration caused by technical malfunctions, and the relative niche knowledge required to resolve the issues can perpetuate a reluctance to employ e-learning approaches.

Regardless of individuals’ willingness to participate, online learning events will continue. As such, understanding the related educational theory is critical. Multimedia learning revolves around the idea of learning from words and pictures, with two of the five types of cognitive processes being influenced by the delivery of content, such as the presentations and videos of virtual events. Selecting words refers to the recognition of important spoken phrases by a learner. Selecting images refers to the recognition of important printed words and text [22]. Many of the speakers at our event focused on the inclusion of pictures in their presentations. There is evidence to support the argument that learning becomes more concrete when images are used over on-screen text. This assertion provides one possible explanation for the results of our positive post-event feedback. However, it is important to note that feedback was not targeted towards the effectiveness of multimedia learning, therefore, alternative explanations cannot be excluded. An awareness of the benefits and pitfalls of learning through multimedia modalities would prove useful when planning virtual events.

Recommendation for running a virtual event

Our key recommendations for future event teams based on our experience can be stratified into planning, event promotion, and critical reflection on obtained feedback.

Planning is an essential component of any event. Ensuring regular email contact with speakers prior to the event and reaffirming their availability is crucial. If for any reason they are not available, offering the opportunity to record their talk will enable a contingency plan should any technical issues arise. Event sponsor videos may serve as a suitable alternative to pre-recorded presentations if none are available. A practice simulation of the event with the team in the days before is recommended. In our example, doing so highlighted several issues that were rectified, which would have otherwise caused critical problems on the day [13].

The online platform offers a greater probability of getting renowned educators to speak; however, these people will be busy so giving plenty of notice is vital. The shift online has resulted in a huge influx of competing for virtual events, and now more than ever strong advertisement with unique features is required to stand out. Using all available channels to disseminate the event and ensure to continue marketing as the event approaches. Using a sign-up form such as Google Forms as opposed to a direct Zoom link also minimizes disruption due to the presence of bots/uninvited guests [9]. Expect a significantly higher sign-up than the number of delegates on the day, as attrition from online events is much easier due to the relatively low commitment required for attendance. Consideration should be given to charging a refundable attendance fee, as a means to increase the commitment required of delegates. This should be balanced against the risk of putting off individuals who may struggle to afford such costs. The decision should be made in the context of your event, including the size, training level of attendees, and content.

**Figure 3:**
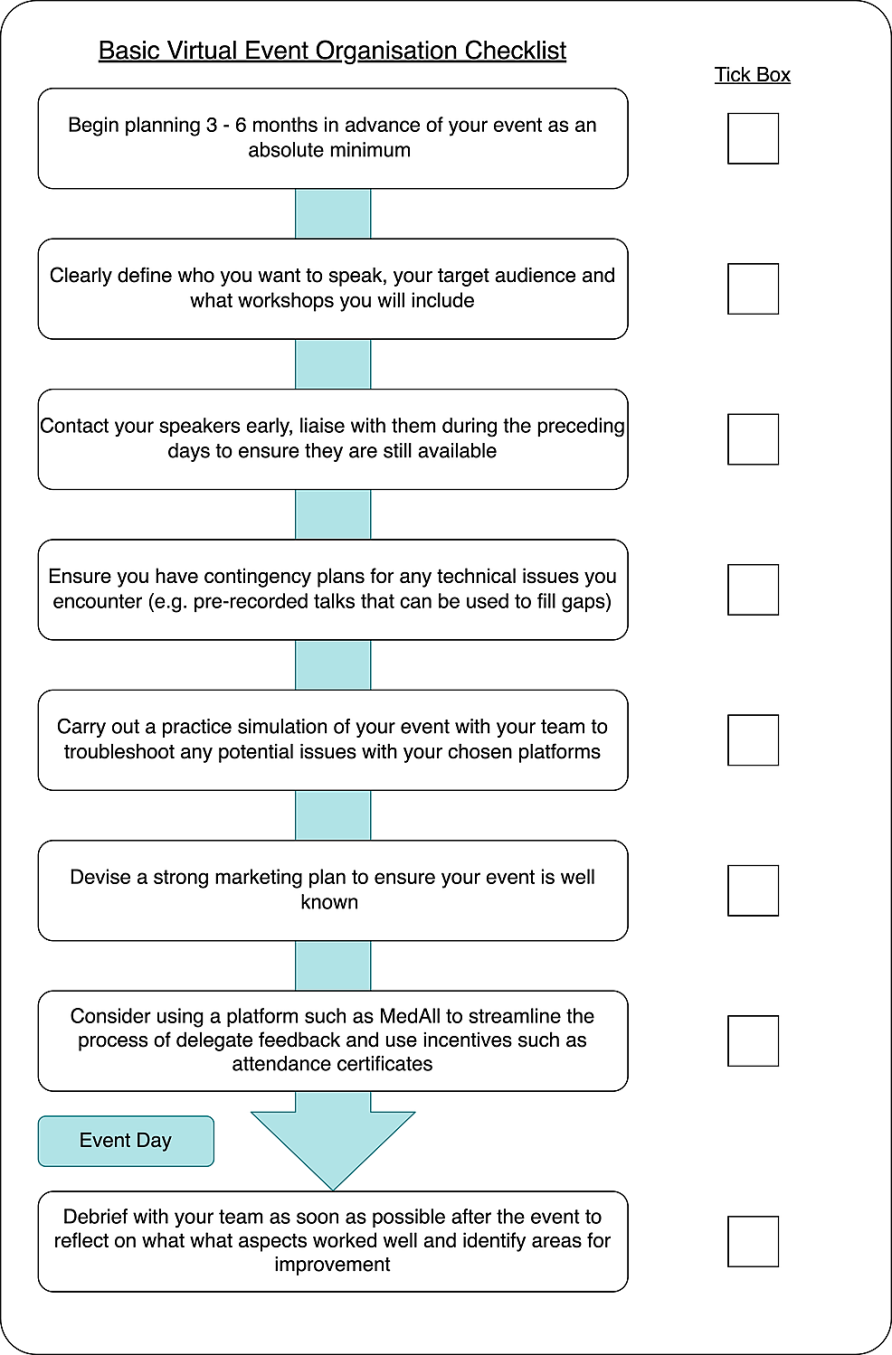
Key steps checklist for hosting a virtual event.

Finally, appreciating that mistakes are inevitable, and carrying out a team debrief session post-event to reflect on what went well and highlight areas for improvement. Ensuring the completion of all feedback is imperative. This can be streamlined by using platforms such as MedAll, Hopin, and others. These incentivize feedback completion by automating the issuing of attendance certificates. Figure 3 is a checklist that we have devised and features our summarised, transferrable recommendations for a virtual event.

Limitations

The results of this project are subject to two key limitations. One limitation is the use of project-specific questions for feedback forms. The use of unvalidated questions may have resulted in a degree of bias when responding to the delegate survey. However, it was felt that due to the niche nature of the neurosurgery careers day, using specific questions would result in a higher degree of granularity within the data. A second limitation of this project is the generalizability of the results. Compared to previous careers days, there was a large increase in the variation of delegate nationality. Despite this, the vast majority of delegates attended from the UK. As such, the conclusions drawn from attendee feedback may be limited, and therefore may not apply as strongly to international medical education.

## Conclusions

The experience of hosting the 10th Annual NANSIG-SBNS Neurosurgery Careers Day has highlighted the feasibility of running a successful online education event. Within this article, we have discussed the key themes encompassed by the day in the context of our delegate feedback. The shift to the virtual model has had a multitude of implications for attendance, teaching, and networking -- some of which are beneficial whilst others are deleterious in nature. We have proposed a transferable framework for hosting online events that can be easily applied to student-led events. The future of online events is unclear and COVID-19 vaccines provide the potential for a return to in-person events. The positives of virtual events, ease of participation, engagement, and accessibility, should not be disregarded. We recommend a hybrid approach henceforth.

## Appendices

Appendix 1 

**Table 3:** Pre-event delegate questionnaire. Questionnaire was completed using Google Forms.

Question Number	Question	Response format
1	What is your stage in training?	Choice from: Year 1 medical student, Year 2 medical student, Year 3 medical student, Year 4 medical student, Year 5 medical student, Year 6 medical student, Intercalating medical student, doctor, neurosurgical trainee, other (free text)
2	What country are based in?	Free text
3	How interested are you in a career in neurosurgery?	5 Point Likert Scale
4	How did you hear about the careers day?	Choice from: social media, medical school, hospital trust, friends/family, NANSIG website, work colleagues, other (free text)
5	I feel inspired to pursue a career in neurosurgery	5 Point Likert Scale
6	I have attended a virtual neurosurgery conference before	Choice from: yes, no
7	My knowledge of neurosurgery as a career is…	5 Point Likert Scale
8	My knowledge of the neurosurgical application process in the UK is…	5 Point Likert Scale
9	I think women are well represented in neurosurgery events	5 Point Likert Scale
10	I think ethnic minorities are well represented in neurosurgery events	5 Point Likert Scale
11	I think virtual neurosurgery careers days are not as good as in-person ones	5 Point Likert Scale
12	There is not much chance to network at virtual neurosurgery events	5 Point Likert Scale

Appendix 2 

**Table 4:** Post-event delegate feedback questions. Questionnaire was completed using the MedAll online platform. Once completed event attendance certificates were issued.

Question Number	Question	Response format
1	What is your stage in training?	As in Appendix 1
2	What country are based in?	As in Appendix 1
3	After attending the careers day, how interested are you in a career in neurosurgery?	5 Point Likert Scale
4	I feel inspired to pursue a career in neurosurgery	5 Point Likert Scale
5	Was the virtual careers day well organized?	Choice from: yes, no, neither
6	After attending the careers day, my knowledge of neurosurgery as a career is…	5 Point Likert Scale
7	What could have been better?	Free text
8	Which CV clinic did you go to, and how was it?	Choice of CV clinic and free text
9	After attending the careers day, my knowledge of the neurosurgical application process in the UK is…	5 Point Likert Scale
10	I think women were well represented at the NANSIG careers day	5 Point Likert Scale
11	I think ethnic minorities were well represented at the NANSIG careers day	5 Point Likert Scale
12	I think virtual neurosurgery careers days are not as good as in-person ones	5 Point Likert Scale
13	There is not much chance to network at virtual neurosurgery events	5 Point Likert Scale
14	The speakers and program was well organized	5 Point Likert Scale
15	The conference program was diverse and catered to a variety of interests	5 Point Likert Scale
16	I enjoyed the MedAll poster presentations gallery	5 Point Likert Scale
17	I would attend another virtual neurosurgery careers day from NANSIG	5 Point Likert Scale
18	I would be interested in attending future NANSIG events	5 Point Likert Scale
19	Please give any overall comments, or feedback on the careers day	Free text
